# Supplementary material for: Evolutionary Quantitative Genomics of Populus trichocarpa
Source: PLoS One. 2015 Nov 23;10(11):e0142864. doi: 10.1371/journal.pone.0142864 (PMC4658102; doi:10.1371/journal.pone.0142864)
Supplement: S1 Fig — (a) F ST outlier loci detection and distribution of empirical F ST estimates conditioned on expected heterozygosity (HE). The envelope of values corresponding to neutral expectations at 99% CI level (with mean F ST = 0.0078), solid line, was constructed with the infinite allele model according to [9]. (b) Distribution of the empirical F ST estimates along the 19 poplar chromosomes and additional scaffolds (abbrev: scaff); the 121 identified outlier loci are indicated by red circles above their F ST value bars. A goodness-of-fit test assuming a uniform distribution was performed to test whether the observed frequencies of ‘outlier loci’ along the 19 poplar chromosomes differed significantly from the expected value. Following the rejection of the null hypothesis (chi-square = 81.98 df = 18, P-value = 3.85e-10), we declared ‘outlier loci hotspots’ if the number of outliers at a given chromosome was equal or above the maximum value (i.e., 20) for assessed outlier clusters from a randomly generated data set using the 118 outliers found across the 19 chromosomes, and running 1,000 replicates, which identified significant clustering of outliers on chromosome 15. (PDF) [file pone.0142864.s001.pdf]

(a)

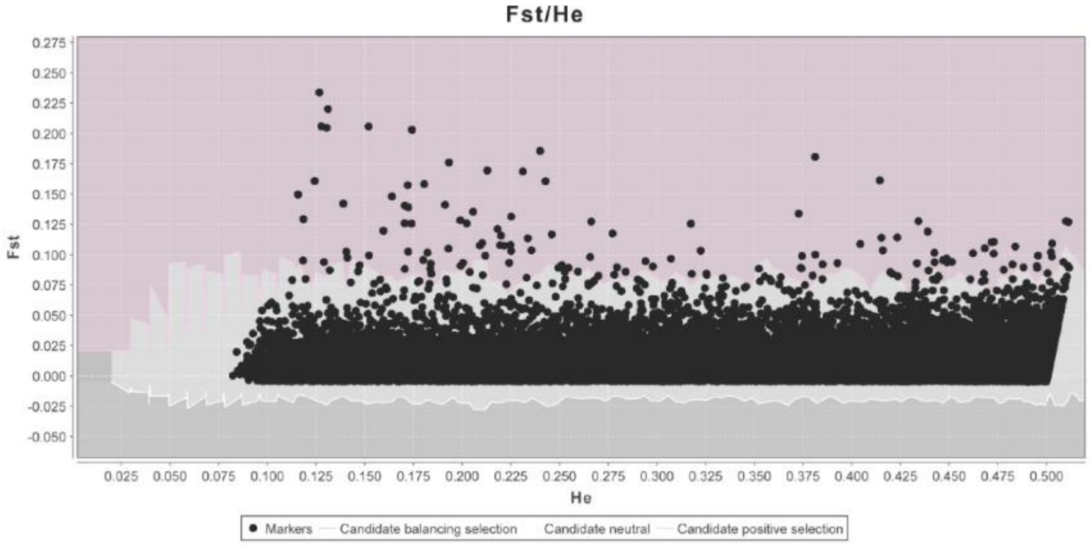

(b)

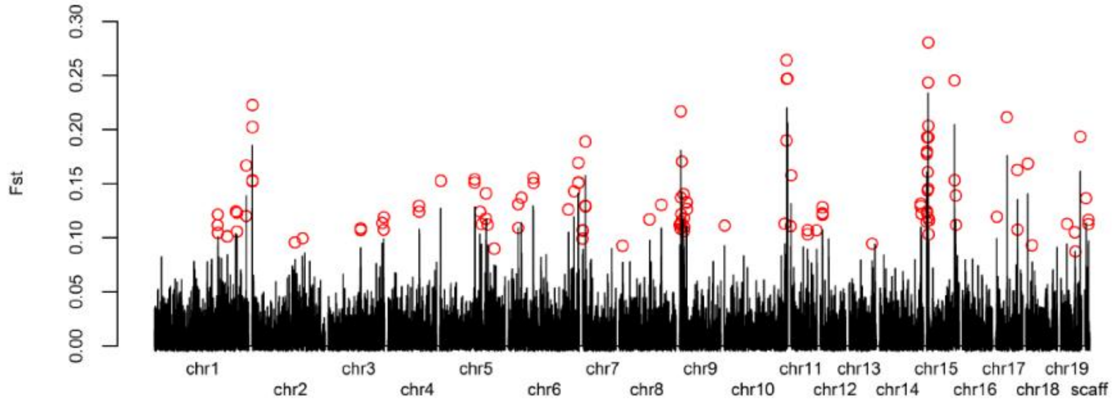

Fig. S1. F_{ST} outlier loci detection in *P. trichocarpa* and distribution of outliers along the poplar chromosomes.

Caption: (a) F_{ST} outlier loci detection and distribution of empirical F_{ST} estimates conditioned on expected heterozygosity (H_E).

The envelope of values corresponding to neutral expectations at 99% CI level (with mean F_{ST}=0.0078), solid line, was constructed with the infinite allele model according to (Beaumont & Nichols, 1996) (b) Distribution of the empirical F_{ST} estimates along the 19 poplar chromosomes and additional scaffolds (abbrev: scaff); the 121 identified outlier loci are indicated by red circles above their F_{ST} value bars.

A goodness-of-fit test assuming a uniform distribution was performed to test whether the observed frequencies of 'outlier loci' along the 19 poplar chromosomes differed significantly from the expected value. Following the rejection of the null hypothesis (chi-square = 81.98 df = 18, *p*-value = 3.85e-10), we declared 'outlier loci hotspots' if the number of outliers at a given chromosome was equal or above the maximum value (*i.e.*, 20) for assessed outlier clusters from a randomly generated data set using the 118 outliers found across the 19 chromosomes, and running 1,000 replicates, which identified significant clustering of outliers on chromosome 15.
