# Supplementary material for: Evolutionary Quantitative Genomics of Populus trichocarpa
Source: PLoS One. 2015 Nov 23;10(11):e0142864. doi: 10.1371/journal.pone.0142864 (PMC4658102; doi:10.1371/journal.pone.0142864)
Supplement: S2 Fig — Simple linear regression (R2) of allelic frequencies (following arcsine transformation) on temperature and precipitation, respectively (mean annual temperature in °C: MAT_1971–2002; number of frost-free days: NFFD_1971–2002 and mean annual precipitation in mm: MAP_1971–2002, observed between yrs 1971–2002) calculated among the four distinct climate clusters (Fig 1); Note: POPTR_0143s00200 was recently re-annotated to Potri.009G008500 and both genes are now assembled on chromosome 9 within 50kb of each other (new poplar genome assembly Phytozyme v3). Both sequences are now described as tandem gene pair PTNRT2.4A (alias Potri.009G008600) and PTNRT2.4B (alias Potri.009G008500) with 97% DNA sequence similarity [62]. (PDF) [file pone.0142864.s002.pdf]

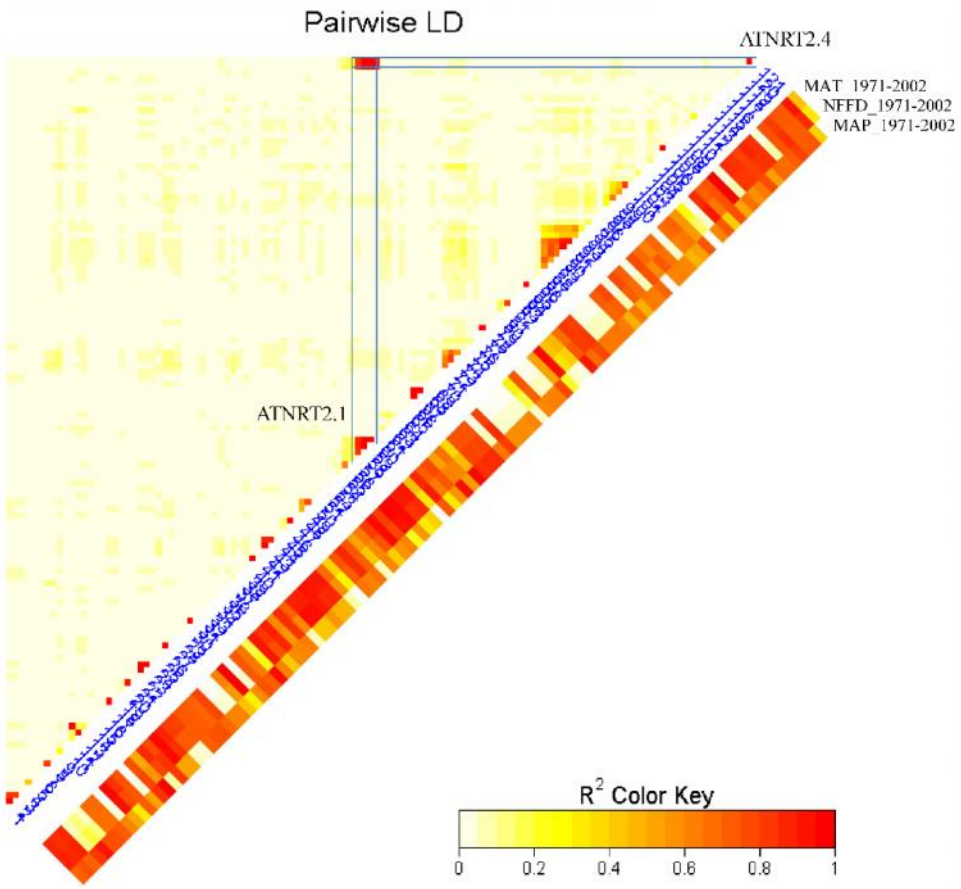

Fig. S2. Linkage disequilibrium between 121 identified F_{ST} outlier loci and relationship between F_{ST} outlier allele frequencies and climate variables in *P. trichocarpa*.

Simple linear regression (R^2) of allelic frequencies (following arcsine transformation) on temperature and precipitation, respectively (mean annual temperature in °C: MAT_1971-2002; number of frost-free days: NFFD_1971-2002 and mean annual precipitation in mm: MAP_1971-2002, observed between yrs 1971-2002) calculated among the four distinct climate clusters (Fig. 1); Note: POPTR_0143s00200 was recently re-annotated to Potri.009G008500 and both genes are now assembled on chromosome 9 within 50kb of each other (new poplar genome assembly Phytozyme v3). Both sequences are now described as tandem gene pair *PTNRT2.4A* (alias Potri.009G008600) and *PTNRT2.4B* (alias Potri.009G008500) with 97% DNA sequence similarity (Bai *et al.*, 2013).

The order of loci follows:

1 scaffold_1_27485620
2 scaffold_1_27487874
3 scaffold_1_27488119
4 scaffold_1_33628533

19	5	scaffold_1_33632379
20	6	scaffold_1_37065304
21	7	scaffold_1_37410840
22	8	scaffold_1_37410856
23	9	scaffold_1_45757179
24	10	scaffold_1_45758739
25	11	scaffold_2_127966
26	12	scaffold_2_128416
27	13	scaffold_2_128432
28	14	scaffold_2_130506
29	15	scaffold_2_10949533
30	16	scaffold_2_13035475
31	17	scaffold_3_14135487
32	18	scaffold_3_14135542
33	19	scaffold_3_19339785
34	20	scaffold_3_19747482
35	21	scaffold_3_19750521
36	22	scaffold_4_17161026
37	23	scaffold_4_17161413
38	24	scaffold_4_17162655
39	25	scaffold_5_88127
40	26	scaffold_5_12339685
41	27	scaffold_5_12344723
42	28	scaffold_5_16487025
43	29	scaffold_5_16811923
44	30	scaffold_5_19211088
45	31	scaffold_5_19211834
46	32	scaffold_5_19953723
47	33	scaffold_5_22633044
48	34	scaffold_6_2485373
49	35	scaffold_6_2489698
50	36	scaffold_6_3249232
51	37	scaffold_6_6390362
52	38	scaffold_6_6436509
53	39	scaffold_6_23299767
54	40	scaffold_6_24631540
55	41	scaffold_6_24634215

42	scaffold_6_25893186
43	scaffold_6_25893407
44	scaffold_6_25893900
45	scaffold_7_74879
46	scaffold_7_178643
47	scaffold_7_179188
48	scaffold_7_808919
49	scaffold_7_809632
50	scaffold_7_811143
51	scaffold_8_805284
52	scaffold_8_6567373
53	scaffold_8_9267412
54	scaffold_9_1379696
55	scaffold_9_1599746
56	scaffold_9_1606213
57	scaffold_9_1676227
58	scaffold_9_1676590
59	scaffold_9_1678624
60	scaffold_9_1678826
61	scaffold_9_2160922
62	scaffold_9_2563600
63	scaffold_9_2677917
64	scaffold_9_2679340
65	scaffold_9_2687811
66	scaffold_9_3795784
67	scaffold_9_3798176
68	scaffold_9_3800384
69	scaffold_10_255159
70	scaffold_10_20168770
71	scaffold_10_21246081
72	scaffold_10_21249991
73	scaffold_10_21253673
74	scaffold_10_21451968
75	scaffold_11_145058
76	scaffold_11_295988
77	scaffold_11_15084939
78	scaffold_11_15084942

93	79	scaffold_11_18477497
94	80	scaffold_12_1811250
95	81	scaffold_12_1811719
96	82	scaffold_12_1812031
97	83	scaffold_13_14296993
98	84	scaffold_14_12173467
99	85	scaffold_14_12173560
100	86	scaffold_14_12927245
101	87	scaffold_15_133408
102	88	scaffold_15_247054
103	89	scaffold_15_247527
104	90	scaffold_15_247811
105	91	scaffold_15_267849
106	92	scaffold_15_268612
107	93	scaffold_15_342410
108	94	scaffold_15_382827
109	95	scaffold_15_512479
110	96	scaffold_15_630677
111	97	scaffold_15_703349
112	98	scaffold_15_704562
113	99	scaffold_15_718240
114	100	scaffold_15_719540
115	101	scaffold_15_719682
116	102	scaffold_15_910808
117	103	scaffold_15_1006871
118	104	scaffold_15_13596400
119	105	scaffold_15_13618770
120	106	scaffold_15_13808656
121	107	scaffold_15_13808709
122	108	scaffold_15_13889772
123	109	scaffold_17_724384
124	110	scaffold_17_5220579
125	111	scaffold_17_12392905
126	112	scaffold_17_12436896
127	113	scaffold_18_1110947
128	114	scaffold_18_2565040
129	115	scaffold_19_5985766

130	116	scaffold_19_12221032
131	117	scaffold_19_12484019
132	118	scaffold_19_15299925
133	119	scaffold_21_280997
134	120	scaffold_143_2955
135	121	scaffold_143_3026
